# Longevity Effect of Liuwei Dihuang in Both *Caenorhabditis Elegans* and Aged Mice

**DOI:** 10.14336/AD.2018.0604

**Published:** 2019-06-01

**Authors:** Weidong Chen, Jinzeng Wang, Jiahao Shi, Xu Yang, Ping Yang, Ning Wang, Sai Yang, Tianpei Xie, Hua Yang, Mengjie Zhang, Haiyun Wang, Jian Fei

**Affiliations:** ^1^School of Life Science and Technology, Tongji University, Shanghai 200092, China; ^2^Shenqi Institute for Ethnomedicine, Tongji University, Shanghai 200092, China; ^3^School of Medicine, Tongji University, Shanghai 200092, China; ^4^Shanghai Engineering Research Center for Model Organisms, SRMOC/SMOC, Shanghai 201203, China; ^5^Standard Testing Lab (Shanghai) Co., Ltd., Pudong, Shanghai 201203, China

**Keywords:** traditional Chinese medicine, lifespan, model organisms, microarray, signal pathway

## Abstract

Liuwei Dihuang (LWDH), a famous traditional Chinese medicine, is widely used in the clinical treatment of aging-related diseases in China. However, its pharmacological mechanisms are not clear. In the present study, we evaluated the lifespan extension effect of LWDH in *C. elegans* and mice and revealed its underlying mechanisms. The results showed that LWDH significantly extended the lifespan of *C. elegans* in a dose-dependent manner. LWDH also conferred protection to nematodes against oxidative stress and reduced their fat storage. Genetics analysis and microarray data showed that the longevity effect of LWDH was attributed to the regulation of the innate immune response, proteolysis, lipid metabolism, and the oxidation-reduction process and was dependent on *daf-16.* Among the six herbs in the formula, Radix Rehmanniae Preparata and Fructus Macrocarpii contributed most to the longevity effect of this medicine, while the other four components had a synergistic effect on the longevity effect of the prescription. The lack of any single herb reduced the efficacy of the complete formula. LWDH also extended the lifespan and reduced both the weight and oxidant stress status in aged mice. Taken together, these results suggested that LWDH might function in a multi-target manner to extend the lifespan in both *C. elegans* and aged mice, and the best effect was achieved with the complete formula.

Liuwei Dihuang (LWDH, in the form of a decoction or concentrated pill) is one of the well-known ancient traditional Chinese medicine (TCM) formulas and is composed of six herbs including Radix Rehmanniae Preparata, Fructus Macrocarpii, Rhizoma Dioscoreae Oppositae, Poria, Cortex Moutan Radicis and Rhizoma Alismatis at a weight ratio of 8:4:4:3:3:3. LWDH was first recorded in the book of “Xiaoer YaoZheng ZhiJue” by Yi Qian in 1114 AD, a medical book for the treatment of pediatric diseases. LWDH was originally used for treating children with growth retardation. LWDH is now widely used for treating adults with symptoms related to aging, such as weakness, poor memory and dizziness, in the clinic.

Numerous animal studies have been conducted to reveal the therapeutic effects of LWDH. Perry found that LWDH showed an anti-inflammatory effect by reducing serum C-type protein and interleukin 6 (IL-6) in rats [1]. Several other studies have suggested LWDH may function in the nervous system. Spatial learning ability was improved in rats treated with LWDH by increasing neurogenesis in the dentate gyrus [2]. The results from studies with senescence-accelerated mice also showed that LWDH could ameliorate the age-related decline in learning and memory, which was likely attained by the induction of long-term potentiation (LTP) in hippocampal neurons [3]. Clinical data and laboratory studies also suggest that LWDH could ameliorate diabetic symptoms and enhance antioxidant activity [4-6].

LWDH is also considered a drug with anti-aging potential. However, few studies have referred to its pharmacological effect on lifespan extension. Wan evaluated the lifespan extension effect of several TCM formulas, including LWDH, in *C. elegans* and found that this medicine could extend the lifespan, however, its mechanism remains unclear [7].

*C. elegans* has become a popular model for studying aging due to its unique advantages. This organism has a short lifespan and a well-characterized genome, which shows considerable homology with the human genome. Many conserved signaling pathways are shared between humans and nematodes in controlling lifespan [8-10]. By using this model, several compounds, natural substances and clinical drugs, such as mianserin, royal jelly, blueberry and rifampicin, have been demonstrated to have a lifespan extension effect [11-14].

In the present study, we used *C. elegans* as a model organism to determine whether LWDH could extend the lifespan and explored the underlying mechanisms. Moreover, we used the mouse as a model to evaluate whether the same effect existed in mammals.

## MATERIALS AND METHODS

### Animals

The *Caenorhabditis elegans* strains used in the present study were obtained from the Caenorhabditis Genetics Center (CGC) at the University of Minnesota (Minneapolis, MN, USA). The following strains were used: N2 Bristol (wild type), CB1370, *daf-2(e1370)*, CF1038, *daf-16(mu86)* and TJ356, zIs356 [*daf-16p*::*daf-16a/b*::GFP + *rol-6(su1006)*]. They were maintained at 20 °C on solid nematode growth medium (NGM) seeded with *E. coli* OP50.

C57BL/6J and BALB/c mice were used in the lifespan and serum assays, respectively, and these mice were obtained from Shanghai Model Organisms Center, Inc. (Shanghai, China). The C57BL/6J mice were 22-23 months old, and the BALB/c mice were 21 months old. The mice were housed in a specific pathogen-free facility and maintained under a normal 12:12-h light-dark cycle. The mice were provided *ad libitum* access to food and water, and all assays were approved by the Institutional Animal Care and Use Committee (IACUC) at the Shanghai Research Center for Model Organisms.

### Preparation of the LWDH

LWDH pills (Batch No. 12077321) and individual herbs were purchased from Beijing Tong Ren Tang Co., Ltd. (Beijing, China). For *C. elegans*, LWDH pills were dissolved in S-complete medium to generate different concentrations of aqueous solution. The dosage of LWDH for the mice was calculated as follows: LWDH pills are orally administered at 0.072 g/kg per day for humans, therefore, the dosage was equal to 0.432 g/kg and 0.72 g/kg per day (6 and 10-fold as low and high dose, respectively) for mice.

The individual herbs or formulas consisting of five herbs were generated according to the rigid specifications of the Pharmacopeia of the People’s Republic of China, and they were finally dissolved in S-complete medium, similar to the LWDH pills for worms.

### Lifespan assay

The lifespan assay in *C. elegans* was performed as previously described [15]. Age-synchronized nematodes were transferred to a 96-well plate as L1 larvae together with 6 mg/ml OP50 in 120 μl S-complete medium. A total of 15 μl of FUDR was added 48 h after seeding to prevent self-fertilization (0.12 mM final), and 15 μl of different concentrations of LWDH solution was added 20 h later (day 1 of adulthood). The plate was incubated at 20 °C. Wells containing < 8 or > 14 worms were excluded, and approximately 60 worms were used in each group in an individual assay. The lifespan of each worm was recorded every two days on the basis of body movement.

The lifespan assay in aged mice (C57BL/6J) was conducted under normal living conditions. The mice were randomly divided into three groups (9 mice in the control group, 7 mice in the low-dose group and 10 mice in the high-dose group). Water in case groups were replaced with LWDH solution and changed every day. The time of death of each mouse was recorded.

### Food clearance assay

The food clearance assay was performed in liquid medium in a 96-well plate [16]. Then 150 μl of S-complete medium containing 6 mg/ml OP50 and different concentrations of LWDH were added into each well. There were approximately 10 L1 synchronized worms in each well. The plate was maintained at 20 °C for three days. After three days, the plate was placed on a plate shaker for 2 min and the absorbance of each well was measured at 600 nm (OD_600_) by using a UV spectrophotometer (Eppendorf).

### Stress resistance assay

Prior to the stress resistance assay, the worms were treated according to the method described in the lifespan assay, however, for this assay, the worms were transferred into a 25-cm^2^ cell culture flask. After a five-day treatment, the worms were collected and washed three times with M9 buffer to remove both OP50 and LWDH.

For the assay of oxidative stress, approximately 90 worms in each group were transferred into three wells in a 48-well plate containing M9 buffer with 50 mM paraquat or 10 mM K_2_Cr_2_O_7_. The plate was shaken every five hours and the fraction of live nematodes was scored [17].

For the heat stress assay, approximately 90 worms in each group were transferred into three NGM plates. The plates were maintained at 35 °C. The worms were observed every five hours, and the death event was recorded.

### Nile Red staining

The worms were pretreated and collected as described above. Then, the supernatant was removed and 50 μl of freshly prepared 10% paraformaldehyde solution was added. The liquid was treated two times with cyclic liquid nitrogen freezing-thawing. Then, the worms were centrifuged, and the liquid was replaced with 5 μg/ml Nile Red. After 30 min, the worms were washed with M9 buffer and transferred onto a 2% agarose pad for microscopic observation and photography. Image-pro plus 6.0 was used to analyze the fluorescence intensity.

### DAF-16 nuclear translocation quantification

Worms of the TJ356 transgenic strain were pretreated as described above. Prior to the quantification, some worms from the control group were incubated at 35 °C for 2 h and regarded as the positive control. The worms were placed on 2% agarose pads and anesthetized with 10mM sodium azide. The number of DAF-16::GFP particles was counted in the whole worm. Image-pro plus 6.0 was used to analyze the fluorescence intensity.

### Microarray assay and bioinformatics analysis

The worms were prepared as described above in 25-cm^2^ cell culture flasks. Both worms on day 10 and day 22 of adulthood were collected in the control group and the case group treated with 1 mg/ml LWDH. The dead worms were removed, and the remaining worms were stored frozen in liquid nitrogen. All 12 samples were sent to Shanghai Biotechnology Company for whole transcriptome microarray assay.

Total RNA was extracted by using TRIZOL reagent. Then the RNA quality was assessed by using an Agilent Bioanalyzer 2100 and RNA samples with a RIN value of at least 8.0 or higher were subjected to further processing. The total RNA was treated according to the manufacturer’s instructions (Affymetrix 3’ IVT Expression Array) to obtain information for gene expression.

Limma package in R was used to determine the differentially expressed genes (DEGs) according to the normalized gene expression data. Genes with a fold-change of 2 or more and a false discovery rate (FDR) of 0.05 or less were regarded as significantly differentially expressed genes. Then Cluster and TreeView were used to draw heat maps to show the expression pattern of the DEGs [18]. The dynamic expression pattern of DEGs was determined by cluster analysis based on the STEM method [19]. To investigate the function of the selected DEGs, gene ontology (GO) analysis was performed to identify the enriched biological process, and Fisher’s exact test was applied to determine the significant GO categories.

**Table 1:** List of the primers used in the present study.

Gene	Forward Sequence	Reverse Sequence
*sea-1*	ATTGAGAAGAGCCGGAAGCC	TGACACCGTCACGAATGAGG
*skr-9*	TCGACTTGATCGTTGCCTGT	CTGCGTCCTTGTCGGTGAT
*ctl-2*	TCCCAGATGGGTACCGTCAT	GGTCCGAAGAGGCAAGTTGA
*F42A10.6*	TACCAACGGAGTACCATGCC	CTTCCTTGCTCGGGTCATCA
*cpr-1*	AGACTACCACGGAGCTGGAT	TTGGAACAGCGTAGGCAGAG
*ugt-41*	TGCGATCTGCCTTGCATAGT	TACTATCTTGTGCCGGCTGC
*nhr-68*	GTAGGAATGGACAAGACT	CCCGAAGAGGAGATAACTG
*dod-3*	GTGCATATTGTGGAGCTGCG	GTACTTGCACGCTGCGAAAA
*lipl-4*	CTCAAAAAGTGTCGATCTTGAGTT	GATGAGGTGAATTGGCGACC
*vit-5*	TCTGGACTCCCAACCGCA	ATTTGAGCACGGAATGGAAGC
*sod-3*	ATCTACTGCTCGCACTGCTT	TGTTCACGTAGGTGGCATGA
*mtl-1*	TGTGAGGAGGCCAGTGAGA	TTAATGAGCCGCAGCAGTT
*act-4*	GCCACCGCTGCCTCCTCATC	CCGGCAGACTCCATACCCAAGAAG

**Table 2:** LWDH extended the lifespan of *C. elegans* at different concentrations.

Treatment	Mean life span ± SEM (days)	Percentage change	Number of animals	*P* value
Control	20.41 ± 1.010	-	58	-
LWDH-0.5 mg/ml	23.74 ± 0.8559	16.30%	54	0.061
LWDH-1 mg/ml	26.97 ± 1.031	32.09%	58	< 0.001 (***)
LWDH-1.5 mg/ml	28.98 ± 1.321	41.98%	59	< 0.001 (***)
LWDH-2 mg/ml	29.84 ± 1.549	46.19%	51	< 0.001 (***)
LWDH-3 mg/ml	26.85 ± 2.496	31.53%	40	< 0.001 (***)
LWDH-5 mg/ml	7.368 ± 1.157	-63.90%	57	< 0.001 (***)
LWDH-10 mg/ml	3.393 ± 0.2096	-83.38%	56	< 0.001 (***)
50 μM Mianserin	29.06 ± 0.9107	42.35%	51	< 0.001 (***)

Data analyzed from an individual assay. The percentage change was obtained by comparing the mean lifespan of the case group with that of the control group.

### Reverse-transcriptase quantitative PCR (RT-qPCR)

Total RNA was reverse transcribed into cDNA by using the PrimerScript RT reagent Kit with gDNA Eraser (Takara) in accordance with the manufacturer’s instructions. Real-time PCR was conducted with SYBR Premix Ex Taq II (Takara) by using QuantStudio 7 Flex Real-Time PCR System (Applied Biosystems). The *act-4* gene was used as an internal control and the primers sequences were shown in Table 1. For data analysis, the qPCR results were quantified as the fold-change between groups by using the 2^-ΔΔCt^ method.

**Figure 1.**
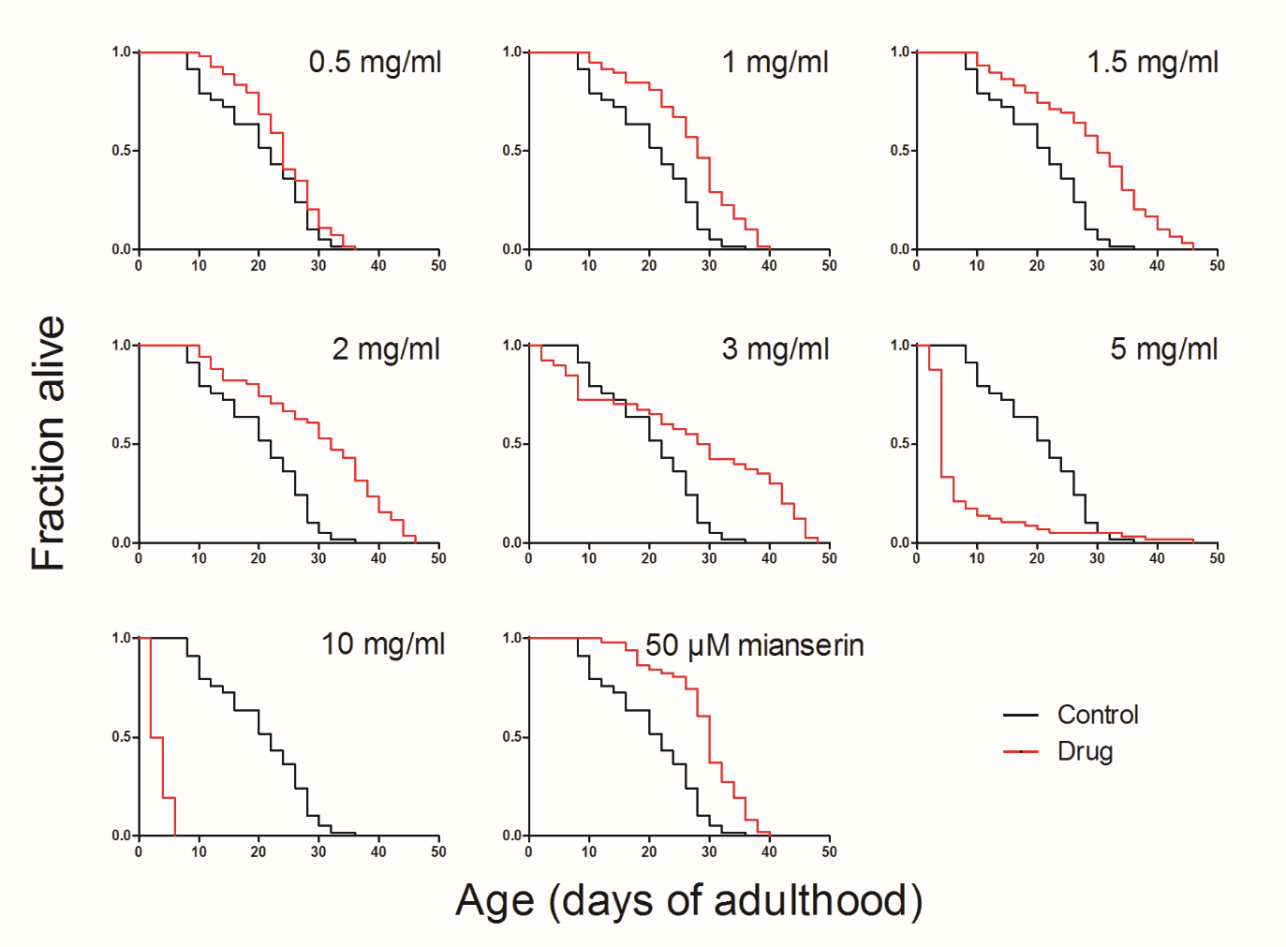
LWDH extended the lifespan of wild-type *C. elegans* at different concentrations LWDH at different concentrations or 50 μM mianserin (positive control) were added into the medium on the first day of adulthood. The survival of worms was monitored every two days until all of the worms were dead.

### Measurement of biochemistry in serum

Aged mice (BALB/c) were fed with low dose of LWDH for 12 weeks, and blood was collected at the end of the assay. Then, the blood was kept at room temperature for two hours and centrifuged at 2000 g for 10 min at 4 °C. The serum was collected and stored at -80 °C. The levels of superoxide dismutase (SOD) and malondialdehyde (MDA) were measured by using commercial kits (Nanjing Jiancheng Bioengineering Institute, Nanjing, China). The SOD activity assay was performed based on the inhibition of superoxide anion generation by the xanthine-xanthine oxidase system, defined as the amount showing a 50% reduction in the absorbance at 550 nm. The MDA content was measured based on a thiobarbituric acid (TBA) reaction. The principle of this method is the spectrophotometric measurement of the color produced during the reaction of TBA with MDA at 535 nm.

### Statistical analysis

The data were expressed as the means ± SEM. GraphPad Prism 6.0 was used to analyze and plot the data. Comparisons were made between animals treated with different concentrations of LWDH or animals with different backgrounds. In the lifespan assay, the significance of the comparison was calculated by the Log-rank (Mantel-Cox) Test, and comparisons for other assays were determined by Student’s t-test unless otherwise noted. The results were considered statistically significant at *P* value < 0.05. The symbols appearing in the figures and tables were defined as NS: no statistical significance, *: *P* < 0.05, **: *P* < 0.01, and ***: *P* < 0.001.

## RESULTS

### LWDH extended the lifespan of C. elegans in a dose-dependent manner

LWDH was added at different concentrations into the medium on the first day that the nematode was entering into adulthood. Mianserin, which has been demonstrated to extend the lifespan of *C. elegans* effectively, was used as the positive control [11]. The results showed that mianserin treatment extended the life expectancy of the nematode by 42.35%, as reported in the literature. LWDH also effectively increased the lifespan of *C. elegans* in a dose-dependent manner (Fig. 1, Table 2, Supplemental Fig. 1). Notably, 1 mg/ml of LWDH increased the lifespan by 32.09% and 2 mg/ml increased the lifespan by 46.19%. However, an early death event was observed when the concentration reached 3 mg/ml. When the concentration was 10 mg/ml, the worms could barely survive. The 1 mg/ml LWDH concentration was used for subsequent experiments because this concentration of LWDH significantly prolonged the lifespan of nematodes without affecting the growth of the nematodes and their food, the OP50 bacteria (Supplemental Fig. 2 and 3).

**Figure 2.**
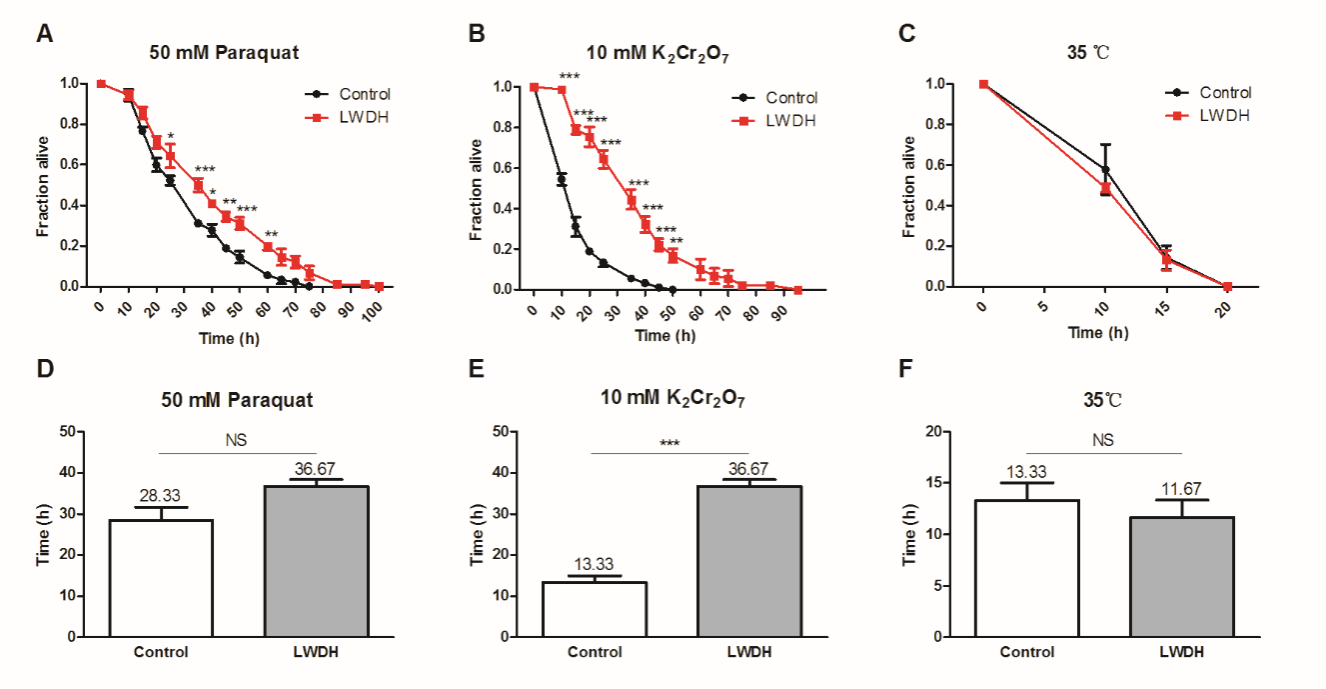
LWDH improved the resistance of *C. elegans* against stress Worms were pretreated with 1 mg/ml LWDH or not for five days. Subsequently, the worms were subjected to oxidative and heat stress. For oxidative stress, the worms were exposed to 50 mM paraquat (**A**) or 10 mM K_2_Cr_2_O_7_ (**B**), and for heat stress, the worms were transferred from 20 °C to 35 °C (**C**). The statistical significance between curves with different time points was calculated by two-way ANOVA. (**D-F**) LT_50_ was calculated and compared between worms pretreated with LWDH or not.

**Figure 3.**
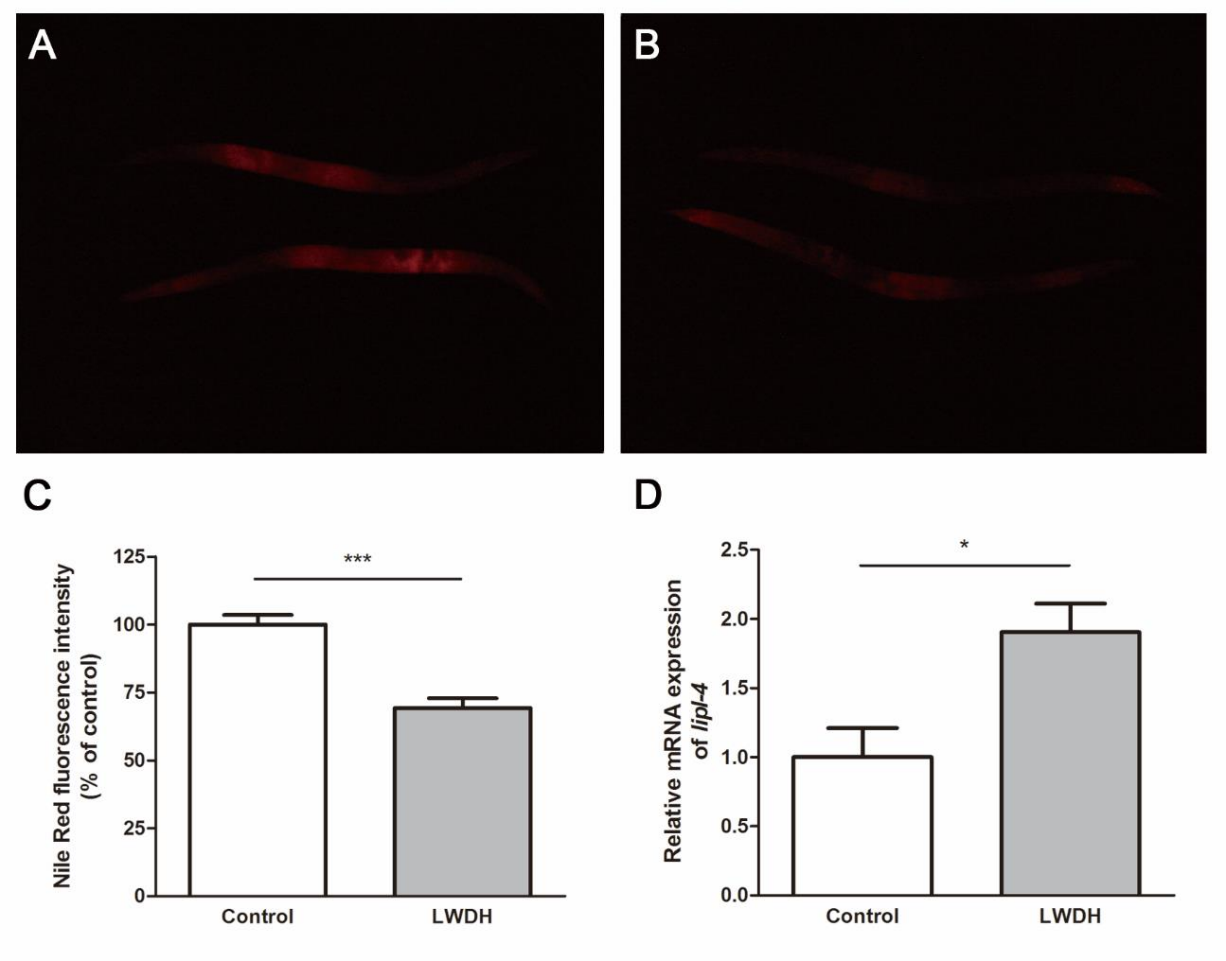
LWDH decreased the fat storage in *C. elegans*. Fat storage was measured with Nile Red after a five-day treatment in the absence (**A**) or presence (**B**) of LWDH. (**C**) Statistical comparisons between the two groups. (**D**) Relative expression levels of *lipl-4* on day 5 after LWDH treatment.

### LWDH improved the stress tolerance in C. elegans

Drugs or genomic modifications that increase the lifespan of nematodes also typically increase their resistance to stresses, such as oxidative stress or heat [20, 21]. Pretreatment with 1 mg/ml of LWDH for 5 days beginning on the first day of adulthood significantly protected the worms against the oxidative stress induced by 50 mM paraquat or 10 mM K_2_Cr_2_O_7_ (Fig. 2A, B). The median lethal time (LT_50_) value demonstrated that LWDH-treated worms showed a 29.44% extension (36.67 h compared with 28.33 h in control worms, *P* = 0.089) against paraquat intoxication, and a more significant extension of 175.10% (36.67 h compared with 13.33 h, *P* < 0.001) against K_2_Cr_2_O_7_ (Fig. 2D, E). However, LWDH treatment did not improve the thermal tolerance of *C. elegans* (Fig. 2C, F).

### LWDH decreased the fat storage in C. elegans

The increase in lifespan may also be accompanied by changes in the metabolism process, such as fat metabolism. In *C. elegans*, reduced insulin/IGF-1signaling (IIS) or loss of the germ line would alter the fat storage [22]. To determine whether LWDH influenced the fat storage, we measured the fat in worms 5 days after treatment with 1 mg/ml LWDH. The fat storage was significantly reduced in worms pretreated with LWDH (Fig. 3A-C).

### The effect of LWDH in aging-related mutants

**Figure 4.**
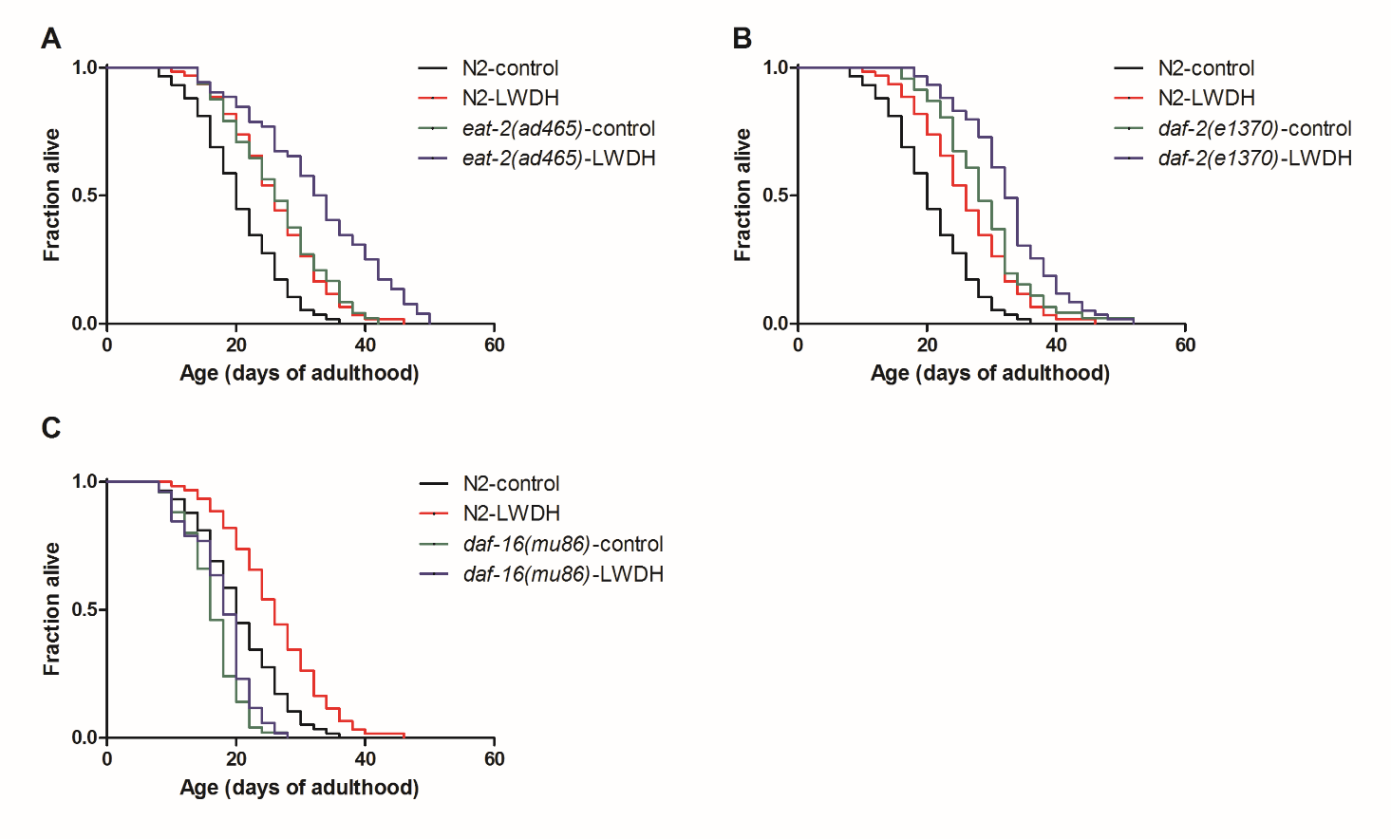
The effects of LWDH in aging-related mutants *eat-2(ad465)* (**A**), *daf-2(e1370)* (**B**), *daf-16(mu86)* (**C**) and wild-type N2 were treated with 1 mg/ml LWDH or not on the first day of adulthood to evaluate the effect of lifespan extension.

To identify the mechanism of LWDH in lifespan extension, we examined the effect of LWDH on several pathways that modulate the lifespan of *C. elegans*. Dietary restriction efficiently extended the lifespan in a wide range of species [23]. The *eat-2(ad465)* strain, with a nonsense mutation in the *eat-2* gene, was long-lived because of the reduced food intake [24]. In the present experiment, the lifespan of *eat-2(ad465)* was longer than that of N2. When *eat-2(ad465)* was treated with 1 mg/ml LWDH, its lifespan was also extended by 23.59%, which was similar to that of N2 (Fig. 4A, Supplemental Table 1). This finding suggested that the lifespan extension of LWDH was not achieved by dietary restriction. The IIS pathway is conserved from yeast to humans. Several genes in the IIS pathway participate in the lifespan-controlling process. In *C. elegans*, *daf-2* is the counterpart to IGF-1 or the insulin receptor in mammals, the mutation of which reduces the IIS pathway and extends the lifespan of the nematode [25]. The present results showed that LWDH prolonged the lifespan of *daf-2* mutants, but the effect of extension was not as good as that of the wild type (13.47% in *daf-2* mutant compared with 26.09% in N2), suggesting that the lifespan extension of LWDH may be not or partly dependent on the IIS pathway (Fig. 4B, Supplemental Table 1).

The DAF-16 FOXO transcription factor is required for several pathways involved in the regulation of lifespan, including the IIS pathway, germline signaling and the AMPK pathway [9, 22, 26-28]. The present results showed that *daf-16(mu86)* mutant worms were short-lived compared with the wild-type worms. After treatment with 1 mg/ml LWDH, the lifespan was extended by 8.33%, which was less than the 26.09% extension observed in N2 worms. Furthermore, this result did not reach statistical significance (Fig. 4C, Supplemental Table 1), suggesting that the lifespan extension of LWDH was mainly dependent on *daf-16*.

### LWDH promoted DAF-16 nuclear localization

**Figure 5.**
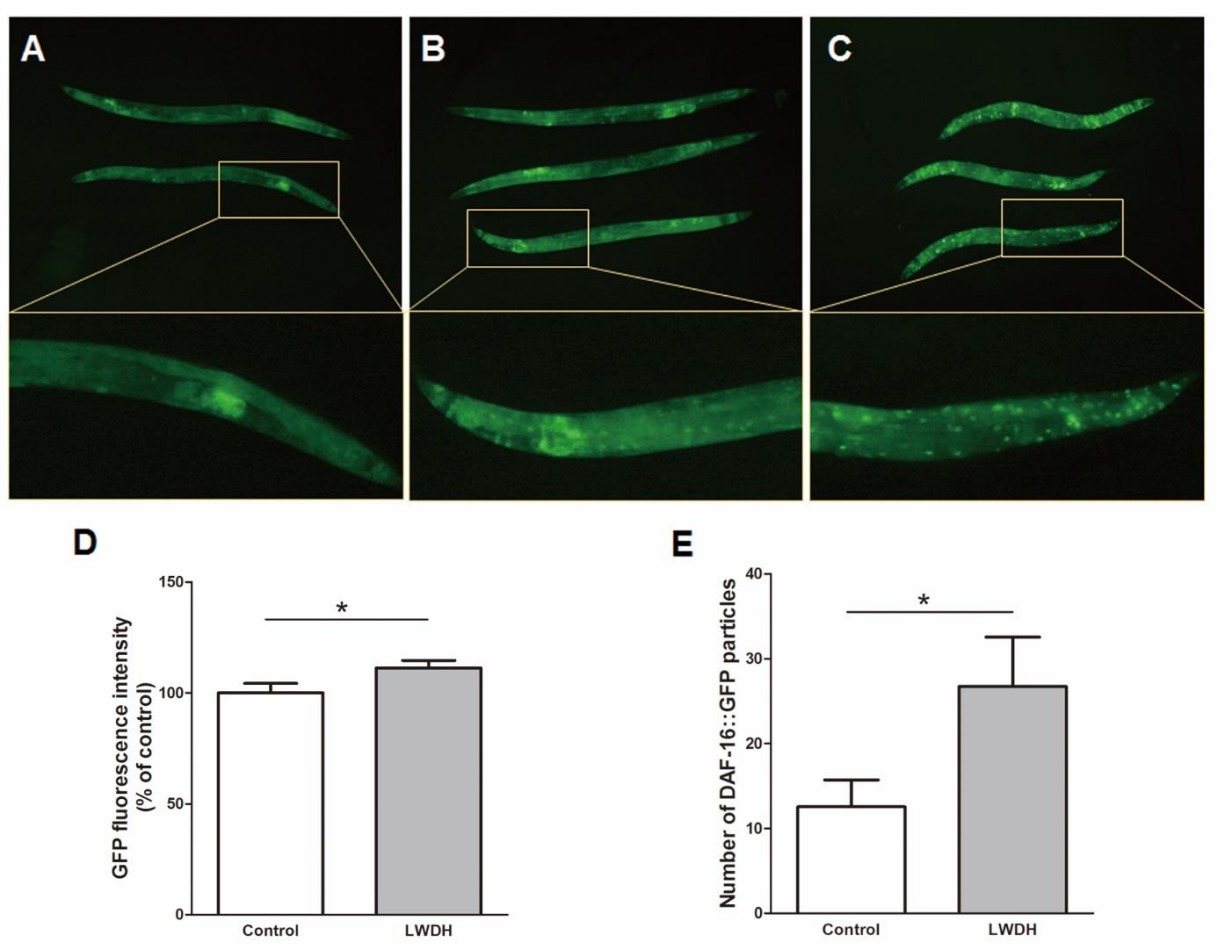
LWDH promoted DAF-16 nuclear localization Representative images for the localization phenotype of DAF-16::GFP in transgenic TJ356 strain after a ten-day treatment in the absence (**A**) or presence (**B**) of LWDH, and the worms maintained at 35 °C for 2 h were regarded as the positive control (**C**). The GFP fluorescence intensity (**D**) and the number of DAF-16::GFP particles (**E**) were measured according to the images.

Since the nuclear localization of the transcription factor is essential for its bioactivity, we examined the translocation of DAF-16 after a ten-day treatment with LWDH. The results showed that LWDH-treated worms displayed significantly increased fluorescence intensity compared with the control, and the number of DAF-16::GFP particles increased (Fig. 5). These results indicated that LWDH increased the level of DAF-16 in worms and stimulated its nuclear translocation.

### LWDH extended the lifespan of C. elegans in a multi-target manner

**Figure 6.**
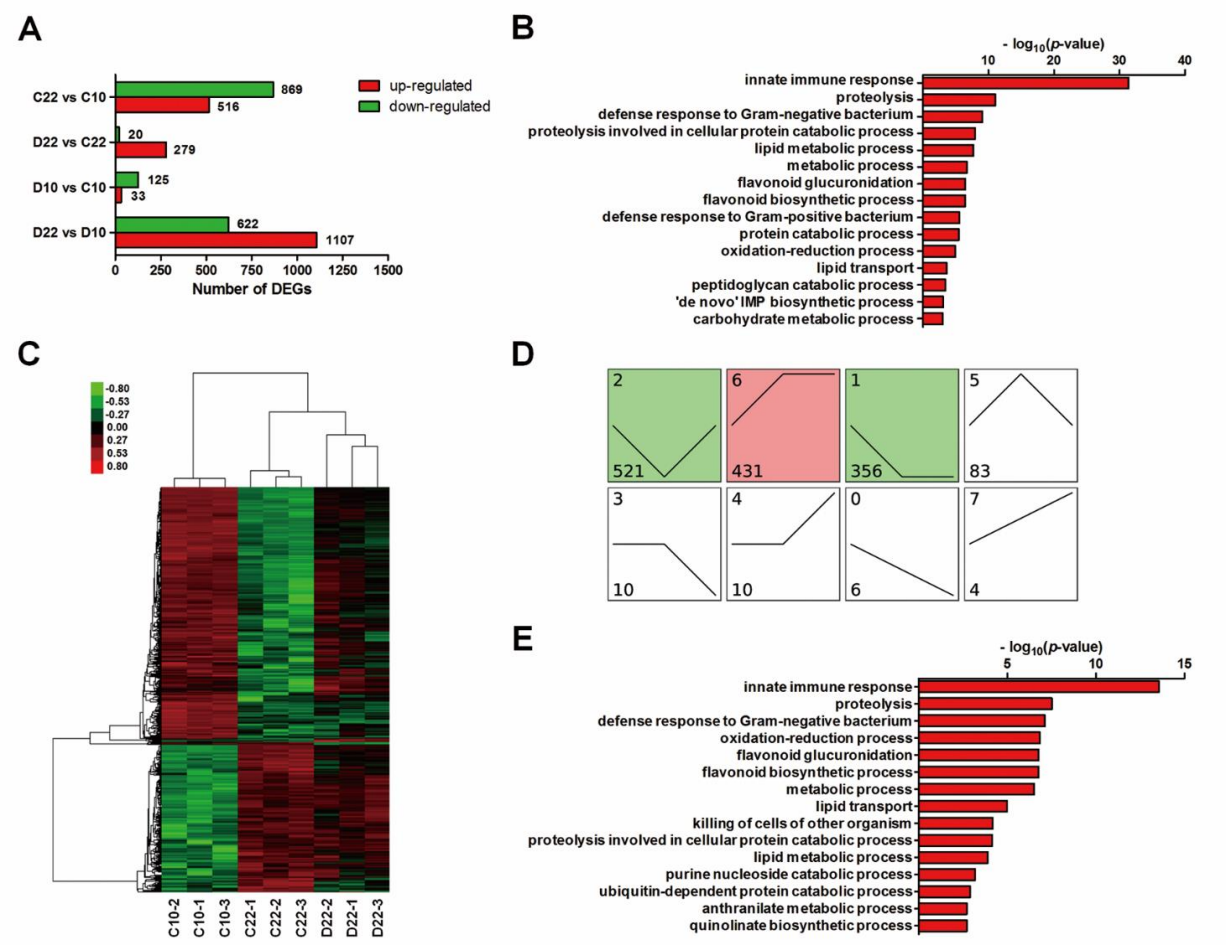
LWDH extended the lifespan of *C. elegans* in a multi-target manner (**A**) The symbol of C and D in the group names represented worms treated with the absence (control) or presence (1 mg/ml) of LWDH, respectively. Days 10 and 22 of adulthood were represented as 10 and 22, respectively. DEGs were analyzed between groups, and numbers of up- or downregulated genes were provided. (**B**) GO analysis was performed with the downregulated genes in C22 vs. C10 and terms of biological process (BP) were shown. (**C**) The heat map showed the expression pattern of DEGs from C22 vs. C10 and D22 vs. C22. Red denoted higher expression level, and green denoted lower expression level. (**D**) Dynamic expression pattern of DEGs from C22 vs. C10 and D22 vs. C22, and genes were classified into 8 clusters. Numbers on top left corner indicated the cluster number. Numbers on bottom left corner indicated the number of DEGs. The colored clusters (1, 2 and 6) indicated significantly enriched ones by DEGs (*P* < 0.05). (**E**) GO analysis showed the BP terms of DEGs from cluster 2.

To further explore the underlying mechanisms of the effect of LWDH on longevity, we performed a whole-transcriptome assay. Worms on days 10 and 22 were regarded as young and aged, respectively, and worms treated with 1 mg/ml LWDH or not were collected. The gene expression levels in these four groups were analyzed and classified into four groups of DEGs (Fold-Change > 2, FDR < 0.05) (Fig. 6A).

Among these genes, 516 genes were upregulated, and 869 genes were downregulated in C22 vs. C10. GO analysis showed that those downregulated genes mainly participated in innate immune response, proteolysis and lipid metabolic process, which indicated that these biological processes were downregulated during aging (Fig. 6B). When we compared the gene expression levels between D22 and C22, we identified 299 DEGs, among which almost all of them (279/299) were upregulated (Fig. 6A). The upregulated genes mainly participated in innate immune response, proteolysis and lipid metabolism. Similar changes in these biological processes were found in the aging process (C22 vs. C10) but in the opposite direction. The downregulation of some genes during aging was reversed by LWDH treatment (Fig. 6C). We merged the DEGs in C22 vs. C10 and D22 vs. C22 to characterize their dynamic expression patterns across 3 conditions, C10, C22 and D22. The data showed that these genes were classified into 8 clusters, and three significant expression clusters (cluster 1, cluster 2, cluster 6) were identified (Fig. 6D). For example, the genes in cluster 2 were downregulated in C22 compared with those in C10, but this effect was reversed in D22. This cluster included most of the DEGs (521 genes), and their expression levels were reversed after treatment with LWDH, which suggested that cluster 2 was related to aging and longevity. Functional analysis further showed that these genes mainly participated in innate immune response, proteolysis, lipid metabolism, and oxidation-reduction process (Fig. 6E). Several genes among these biological processes such as *acdh-1*, *lipl-4*, *cpr-1* and *ctl-2* were demonstrated as important in lifespan extension or as targets of DAF-16 [22, 29, 30]. Some of the genes in cluster 2 were validated by real-time PCR and showed the same expression patterns as observed in the microarray assay (Supplemental Fig. 4).

### Radix Rehmanniae Preparata and Fructus Macrocarpii were the main effective herbs

**Figure 7.**
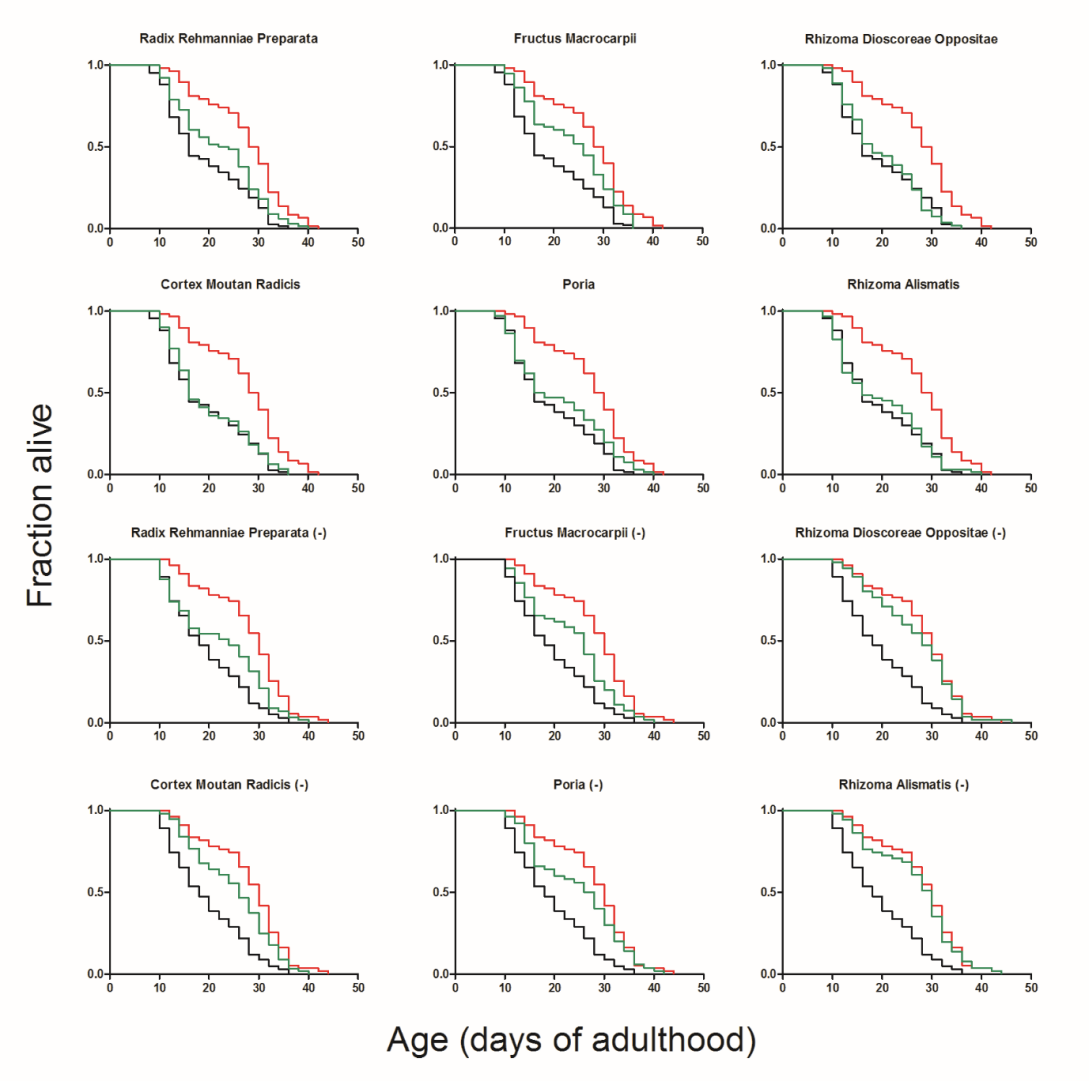
The effects of individual herbs and formulas lacking one herb in wild-type *C. elegans* The complete formula, each individual herb (the first six graphs) or formulas consisting of five herbs (the rest ones, herb with (-) at the end represented its lacking in the formula) were added to the medium on the first day of adulthood. Black lines represented the control group, red lines represented the complete formula, and green lines represented the individual herbs or formulas used. The survival of the worms was monitored every two days until all of the worms were dead.

LDWH is composed of six herbs. We examined the role of each herb in the formula, examined its contribution to the effect of the formulation and determined whether this component is necessary. First, each individual herb was tested for its lifespan extension effect. The complete formula by simply combining the six individual herbs extended the lifespan by 42.61%, which was similar to the effect observed in the former experiment. Among all individual herbs tested, only Radix Rehmanniae Preparata and Fructus Macrocarpii could efficiently extend the lifespan by 15.56% and 22.53%, respectively. However, both of these effects were weaker than those of the complete formula (Fig. 7, Supplemental Table 2). Then, we prepared six new formulas comprising the five herbs. The data showed that all seven formulas could extend the lifespan. However, the effect of the formula lacking Radix Rehmanniae Preparata or Fructus Macrocarpii only prolonged the lifespan by 13.05% and 19.37%, respectively (Fig. 7, Supplemental Table 3). These results indicated that Radix Rehmanniae Preparata and Fructus Macrocarpii were the components that contributed to the formula the most. The other four herbs did not show significant lifespan extension effects individually. However, the lack of each herb weakened the effect of the complete formula, suggesting that these four herbs had synergistic effects in the prescription.

### LWDH exhibited a similar effect in aged mice

**Figure 8.**
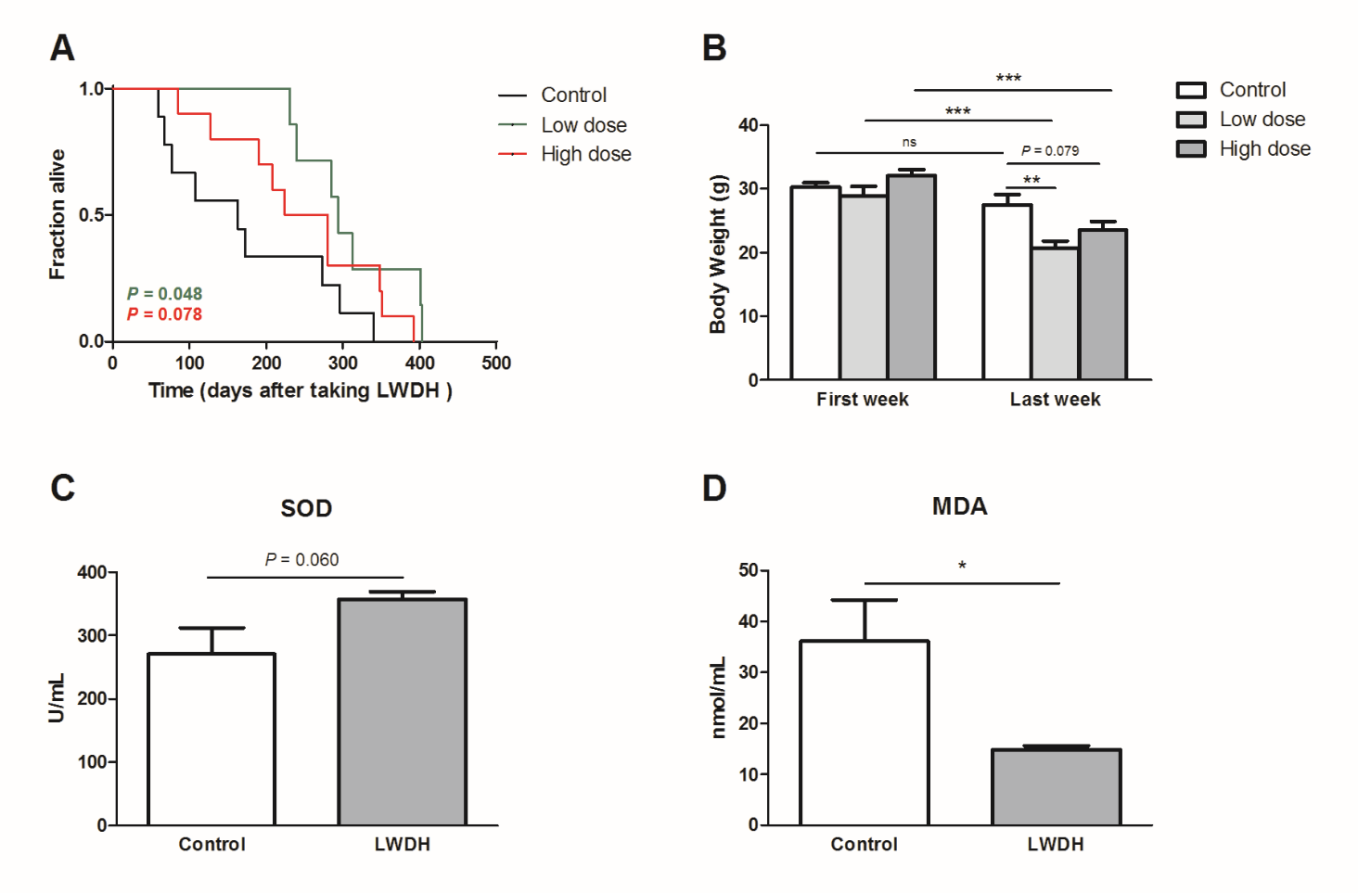
LWDH extended the lifespan of aged mice (**A**) Mice that were 22-23 months old were treated with different doses of LWDH. Day 1 represented the first day of taking LWDH. n = 9, 7, and 10 in control, low-dose and high-dose group, respectively. (**B**) Body weight was measured three times a week and compared between the different groups or between the first and last week in the same group. (**C, D**) The 21-month-old mice were treated with the low dose of LWDH. Serum SOD and MDA were measured after a 12-week treatment. n = 8 in each group.

The 22-23-month-old mice were provided normal drinking water or water supplemented with LWDH. The lifespan was significantly extended in the low-dose group (*P* = 0.048). In the high-dose group, the lifespan was also prolonged, but not significantly (*P* = 0.078) (Fig. 8A). Additionally, the hair of mice in both LWDH-treated groups appeared glossier than that in the control group (data not shown). Furthermore, the weight of the mice was reduced after LWDH treatment compared with that of the control group (Fig. 8B). This finding was consistent with the result that the fat storage was decreased in *C. elegans* after treatment with LWDH, which may be caused by the expression of genes related to fat metabolism.

LWDH can improve the antioxidant capacity of nematodes. We examined whether LWDH could play the same role in mice. The results showed that mice fed LWDH tended to show an increased serum level of SOD (*P* = 0.06), which caused a marked decrease in the serum MDA level, an index of lipid peroxidation (Fig. 8C, D). These results suggested that LWDH also reduced oxidant stress in aged mice.

### DISCUSSION

In this work, we studied the anti-aging effect of LWDH in *C. elegans* and aged mice. The results showed that feeding nematodes and mice with LWDH significantly prolonged their lifespan. In *C. elegans*, this effect was not or partly dependent on IIS pathway and not on dietary restriction. LWDH treatment maintained higher DAF-16 levels and promoted the nuclear localization of DAF-16, and the expression levels of several *daf-16* targets, such as *dod-3*, *ctl-2*, *lipl-4*, *sod-3* and *mtl-1,* were increased on day 22 in the drug-treated group compared with those in the control group (Supplemental Fig. 4). These observations indicated that DAF-16 plays an important role in the lifespan extension effect of LWDH. Additionally, according to the microarray data, the lifespan extension effect of LWDH was associated with a reversal in the expression levels of numerous genes (including the *daf-16* targets) that participate in oxidation-reduction process, lipid metabolism, innate immune response and proteolysis.

Reactive oxygen species (ROS) cause molecular damage within the cell and are widely believed to be one of the primary causes of aging. Experiments in *C. elegans* showed that LWDH protected the nematodes against oxidative stress. Microarray data showed that the expression levels of some antioxidant-related genes were elevated after administration of LWDH, such as *ctl-2,* which encodes a catalase, and *sod-3,* which encodes a superoxide dismutase. Both genes are associated with reducing oxidative damage and play a key role in lifespan extension [29-31]. In LWDH-treated mice, the expression of SOD was also elevated. MDA, an important indicator of tissue damage, was decreased in mice after LWDH administration.

Adipose tissue has been implicated in the regulation of lifespan in several species [32-34]. In *C. elegans*, lipid metabolism prolonged the lifespan, connecting the lipid metabolism function to lifespan control [22]. Several studies have previously demonstrated that LWDH could reduce the body weight and visceral fat in rats [35, 36]. In the present study, we also observed a relationship between the fat storage and lifespan. We found that fat storage was decreased in *C. elegans* and that the body weight of mice declined after LWDH treatment. The *lipl-4* gene encodes a triglyceride lipase whose overexpression could reduce fat storage. In the lifespan assay, *lipl-4* knockdown suppressed the lifespan extension in both *glp-1(e2141)* and *daf-2(e1370)* mutant worms. In contrast, *lipl-4* overexpression in the intestine extended the lifespan in *C. elegans* [22]. In the present microarray data, the expression level of *lipl-4* was decreased during aging and increased on day 22 after LWDH treatment (Supplemental Fig. 4). We then measured the expression level of *lipl-4* on day 5, at which time the fat storage was reduced. RT-qPCR showed that the expression level of *lipl-4* was significantly upregulated by LWDH (Fig. 3D). These data indicated that *lipl-4* may be responsible for the reduced fat storage and extended lifespan of *C. elegans* after LWDH treatment.

Proteostasis and immune system integrity are critical in maintaining a healthy status, and their instability are linked to aging and some age-related diseases [37, 38]. The lysosomal system and the ubiquitin-proteasome system are two principle proteolytic systems. Studies have shown that the activity of proteasomal function declined in aging organisms, and proteasomal-related genes have been proven to be important in longevity [39-42]. The expression of *asp-2*, *cpr-1* and *skr-9*, which encode proteases and components of the ubiquitin ligase complex, was stimulated in the LWDH-treated group. Therefore, proteostasis was improved by LWDH. Living bacterial OP50 is the normal food for worms. However, these bacteria could be pathogenic to worms in an immunocompromised status, and secreted antimicrobial proteins could contribute to the enhanced longevity of *daf-2* mutants [43, 44]. The overexpression of *lys-1* could augment the resistance to *S.marcescens*, and *lys-7* was upregulated in long-lived *daf-2* mutants [29, 43]. In the present microarray data, the expression levels of many genes associated with immune system were upregulated by LWDH, including *lys-1* and *lys-7.*

LWDH is composed of six herbs. According to the theory of traditional Chinese medicine, among the formulation of LWDH, Radix Rehmanniae Preparata is the monarch drug, which is considered to play a major therapeutic role in the formulation. Both Fructus Macrocarpii and Rhizoma Dioscoreae Oppositae are minister drugs, as the assistants of the monarch drug. These three drugs are regarded as the “Three Tonics”. The other three herbs are adjuvant drugs, with roles in reducing side effects and increasing synergy and are regarded as the “Three Purgatives”. Recently, several TCM formulas and individual herbs have been assessed for their therapeutic effects in different disease models [45-47]. Lau found that only the complete formula had the effect on promoting diabetic wound healing, which emphasized the composition principle of TCM [46]. In the present work, we investigated the effect of individual herbs. The present results showed that the monarch drug Radix Rehmanniae Preparata and the minister drug Fructus Macrocarpii extended the lifespan in *C. elegans* respectively, and other herbs also exhibited synergistic effects in the complete formula, which suggested that these six herbs are best combined according to the theory of TCM.

In the present study, the anti-aging effect of LWDH was demonstrated in both *C. elegans* and mammalian mouse models, and similar pharmacological effects were observed. LWDH regulates multiple biological processes in *C. elegans* to promote longevity. Although some DEGs were already implicated in lifespan regulation, there were still several genes with little evidence of their function. These genes are good candidates that need further investigation in the future. These studies would provide a better understanding of the underlying mechanisms of the therapeutic effects of LWDH and the molecular network of lifespan determination.

### Supplemetary Material

The Supplemenatry material for this article can be found online at: www.aginganddisease.org/EN/10.14336/AD.2018.0604


