# Supplementary material for: Longevity Effect of Liuwei Dihuang in Both Caenorhabditis Elegans and Aged Mice
Source: Aging Dis. 2019 Jun 1;10(3):578–91. doi: 10.14336/AD.2018.0604 (PMC6538212; doi:10.14336/AD.2018.0604)
Supplement: Supplementary file 1 [file AD-10-3-578-s.pdf]

SUPPLEMENTARY DATA

Longevity Effect of Liuwei Dihuang in Both *Caenorhabditis Elegans* and Aged Mice

Weidong Chen[1], Jinzeng Wang[1], Jiahao Shi[1], Xu Yang[3], Ping Yang[4], Ning Wang[1], Sai Yang[1], Tianpei Xie[5], Hua Yang[1], Mengjie Zhang[1], Haiyun Wang[1,*], Jian Fei[1,2,*]

[1]School of Life Science and Technology, Tongji University, Shanghai 200092, China
[2]Shenqi Institute for Ethnomedicine, Tongji University, Shanghai 200092, China
[3]School of Medicine, Tongji University, Shanghai 200092, China
[4]Shanghai Engineering Research Center for Model Organisms, SRMOC/SMOC, Shanghai 201203, China
[5]Standard Testing Lab (Shanghai) Co., Ltd., Pudong, Shanghai 201203, China

© 2018. Chen W et al. Published online at http://www.aginganddisease.org/EN/10.14336/AD.2018.0604

SUPPLEMENTARY DATA

Supplemental table 1. Effects of LWDH in aging-related mutants.

Strain & Treatment	Mean lifespan ± SEM (days)	Percentage change	Number of animals	*P* value
N2-control	20.62 ± 0.8509	-	58	-
N2-LWDH	26.00 ± 0.9467	26.09%	61	<0.001 (***)
eat-2(ad465)-control	26.33 ± 1.087	-	48	-
eat-2(ad465)- LWDH	32.54 ± 1.413	23.59%	52	<0.001 (***)
daf-2(e1370)-control	28.74 ± 1.061	-	46	-
daf-2(e1370)- LWDH	32.61 ± 0.9588	13.47%	59	0.011(*)
daf-16(mu86)-control	16.44 ± 0.5946	-	50	-
daf-16(mu86)- LWDH	17.81 ± 0.6777	8.33%	52	0.058

Supplemental table 2. Effects of individual herbs in wild-type *C. elegans*.

Treatment	Mean lifespan ± SEM (days)	Percentage change	Number of worms	*P* value
Control	19.22 ± 0.7745	-	110	-
Radix Rehmanniae Preparata	22.21 ± 1.055	15.56%	66	0.029 (*)
Fructus Macrocarpii	23.55 ± 1.111	22.53%	58	0.002 (**)
Rhizoma Dioscoreae Oppositae	19.85 ± 1.030	3.28%	54	0.994
Cortex Moutan Radicis	19.77 ± 1.025	2.86%	61	0.602
Poria	20.91 ± 1.148	8.79%	66	0.096
Rhizoma Alismatis	19.72 ± 1.092	2.60%	64	0.758
Complete formula	27.41 ± 1.053	42.61%	58	< 0.001 (***)

Supplemental table 3. Effects of formulas lacking one herb in wild-type *C. elegans*.

Treatment	Mean lifespan ± SEM (days)	Percentage change (vs Control)	Number of worms	*P* value (vs Control)	*P* value (vs Complete formula)
Control	19.62 ± 0.7455	-	101	-	< 0.001 (***)
Radix Rehmanniae Preparata (-)	22.18 ± 1.213	13.05%	57	0.021 (*)	0.003 (**)
Fructus Macrocarpii (-)	23.42 ± 1.127	19.37%	55	0.005 (**)	0.005 (**)
Rhizoma Dioscoreae Oppositae (-)	26.47 ± 1.118	34.91%	55	< 0.001 (***)	0.574
Cortex Moutan Radicis (-)	24.86 ± 1.068	26.71%	56	< 0.001 (***)	0.047 (*)
Poria (-)	24.76 ± 1.280	26.20%	50	< 0.001 (***)	0.177
Rhizoma Alismatis (-)	26.78 ± 1.190	36.49%	51	< 0.001 (***)	0.608
Complete formula	28.00 ± 1.048	42.71%	55	< 0.001 (***)	-

© 2018. Chen W et al. Published online at http://www.aginganddisease.org/EN/10.14336/AD.2018.0604

SUPPLEMENTARY DATA

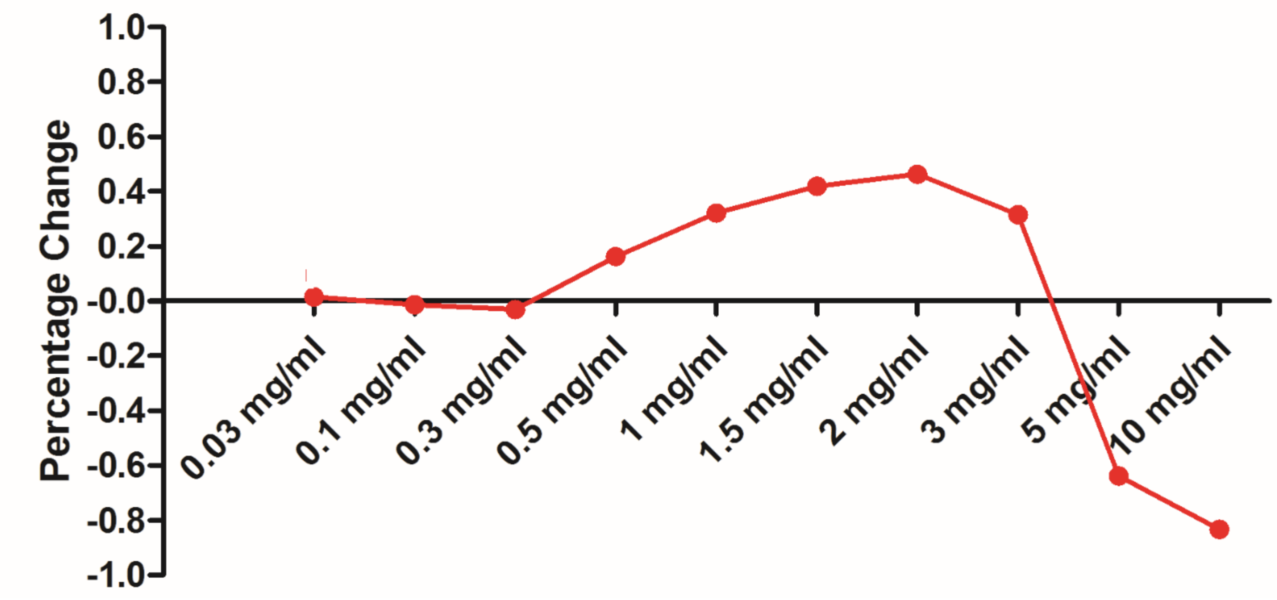

Supplemental Figure 1. Percentage change of lifespan at different concentrations of LWDH. Worms were treated with different concentrations of LWDH from the first day of adulthood. Percentage change of lifespan was obtained by comparing the mean lifespan of LWDH group with that of control group.

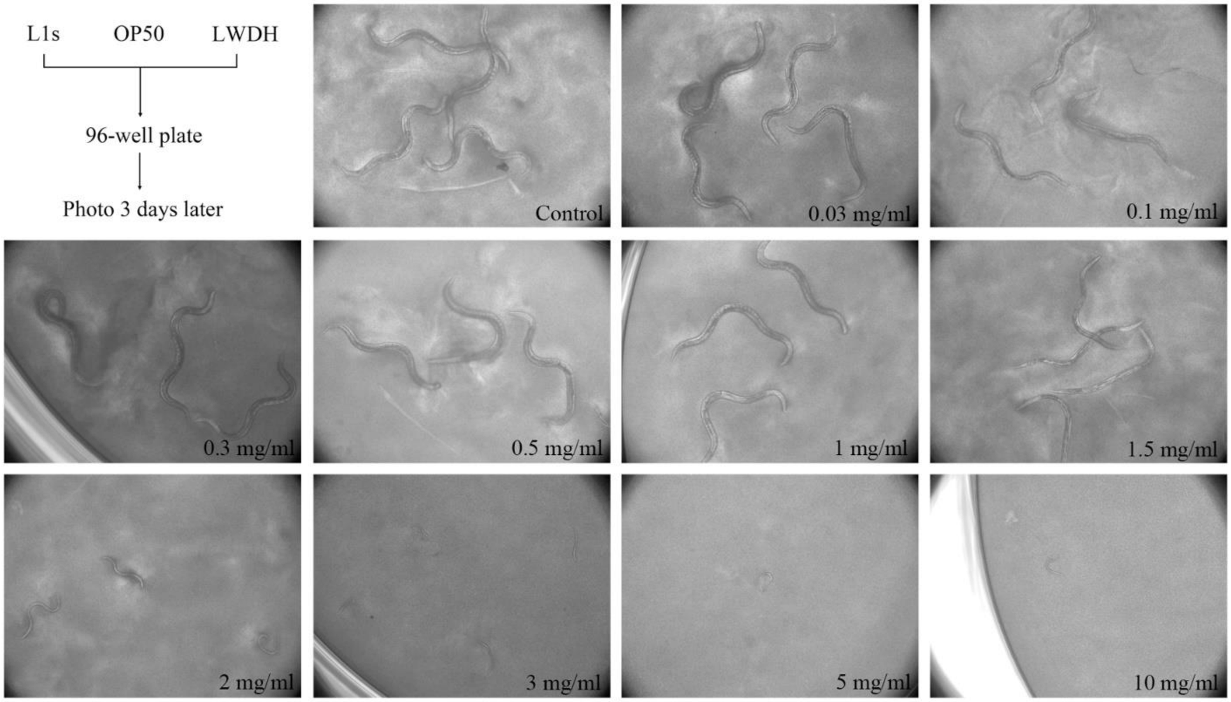

Supplemental Figure 2. Morphological observation of *C. elegans* at different concentrations of LWDH. Top left corner showed the flow diagram of this experiment. Age-synchronized L1s were added into the medium in the presence of both OP50 and different concentrations of LWDH. Developmental status was monitored and photographed three days later. Worms in control group represented the normal developmental status. Low-concentration groups (0.03-1 mg/ml) showed no obviously difference compared with the control group. However, the higher concentration, such as 1.5 mg/ml or even higher ones slowed the normal development. The worms looked younger and there were no eggs in the body.

© 2018. Chen W et al. Published online at http://www.aginganddisease.org/EN/10.14336/AD.2018.0604

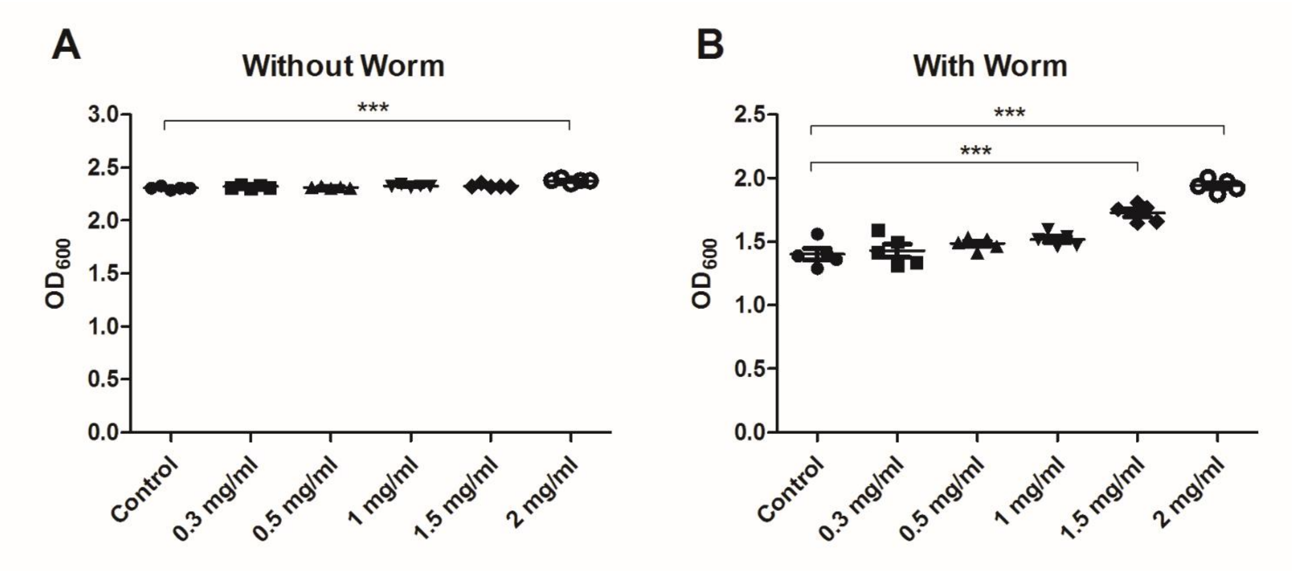

Supplemental Figure 3. Food clearance assay. S-complete containing OP50 and different concentrations of LWDH was added into the 96-well plate, and the plate was maintained at 20 °C for three days. Then the bacterial concentration was obtained by measuring the absorbance at 600 nm in the absence **(A)** and presence **(B)** of worms. After a 3-days co-culturing without worms, OP50 was slightly increased at 2 mg/ml (2.99% higher than control). When the worms were existed in the medium, OP50 was consumed and the remaining amount of OP50 was increased as the concentration raising. The remaining amount of OP50 was significantly higher than control at 1.5 mg/ml and 2 mg/ml.

© 2018. Chen W et al. Published online at http://www.aginganddisease.org/EN/10.14336/AD.2018.0604

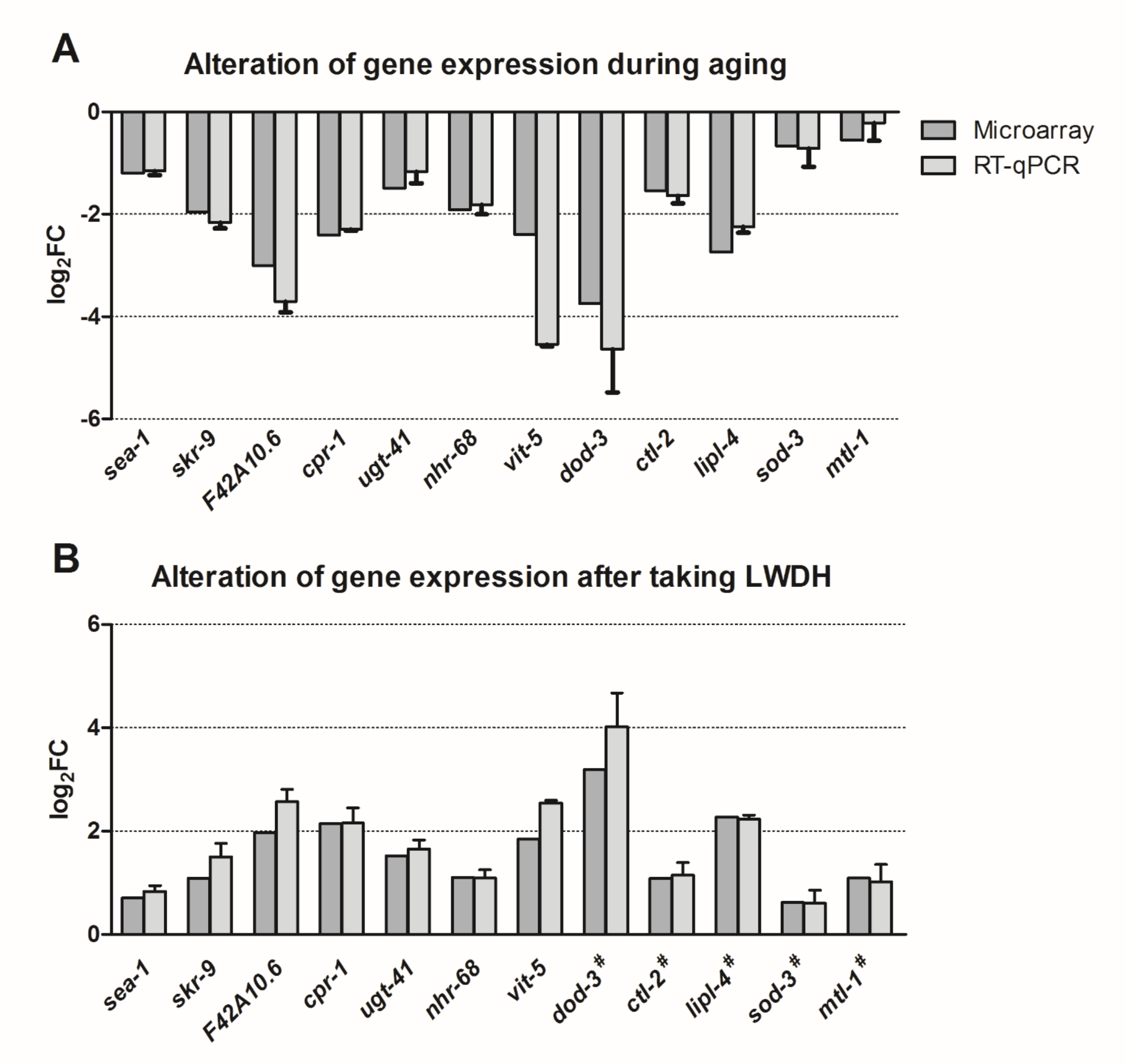

Supplemental Figure 4. RT-qPCR validation. Several genes from cluster 2 or *daf-16* targets (#) were validated by RT-qPCR, *act-4* was used as the reference control. Log2 fold change (log_2FC) reflected the expression patter between groups, the positive value indicated upregulation and the negative value indicated downregulation. Both microarray data and RT-qPCR showed that these genes were downregulated from day 10 to day 22 **(A)** and upregulated after LWDH administration **(B).**

© 2018. Chen W et al. Published online at http://www.aginganddisease.org/EN/10.14336/AD.2018.0604
